# *The Phys-Can observational study*: adjuvant chemotherapy is associated with a reduction whereas physical activity level before start of treatment is associated with maintenance of maximal oxygen uptake in patients with cancer

**DOI:** 10.1186/s13102-020-00205-9

**Published:** 2020-09-03

**Authors:** Tor Helge Wiestad, Truls Raastad, Karin Nordin, Helena Igelström, Anna Henriksson, Ingrid Demmelmaier, Sveinung Berntsen

**Affiliations:** 1 Department of Oncology and Medical Physics, Haukeland University Hospital, Box 1400, 5021 Bergen, PO Norway; 2 Department of Physical Performance, Norwegian School of Sport Sciences, Oslo, Norway; 3 Department of Public Health, Sport and Nutrition, Faculty of Health and Sport Sciences, University of Agder, Kristiansand, Norway; 4 Department of Public Health and Caring Sciences, Uppsala University, Uppsala, Sweden; 5 Department of neuroscience, Uppsala University, Uppsala, Sweden

**Keywords:** Cardiopulmonary exercise testing, Physical activity, Oncological treatment, Cancer related fatigue

## Abstract

**Background:**

Adjuvant therapy may cause multiple sideeffects on long term health, including reduced cardiorespiratory fitness (CRF) in patients with breast cancer (1, 2). However, there is currently limited knowledge regarding the effect of different types of adjuvant cancer treatment on CRF in other cancer populations. The primary objective of the present study was to assess whether previously known correlates (age, diagnosis, initial CRF, physical activity level), type of adjuvant treatment and cancer-related fatigue were associated with changes in $\dot{V}{O}_2\mathit{\max}$ in patients with breast, prostate or colorectal cancer.

**Methods:**

Prospective study with two time points of assessment, 85 patients scheduled for adjuvant cancer treatment were included. Cardiorespiratory fitness was assessed by $\dot{\ V}{O}_2\mathit{\max}$ during a maximal incremental exercise test on a treadmill before start of adjuvant therapy and again six months later. Physical activity level was recorded with a physical activity monitor (Sense Wear™ Mini) at baseline as average minutes of moderate-to-vigorous intensity physical activity (MVPA) per day. Physical fatigue at baseline was reported using the Multidimensional Fatigue Inventory-20 questionaire.

**Results:**

In multivariate linear regression analysis, 30 min higher daily MVPA at baseline was associated with a 5% higher $\dot{V}{O}_2\mathit{\max}$ at six months follow up when adjusted for adjuvant treatment (*P* = 0.010). Patients receiving adjuvant chemotherapy had a mean decline in $\dot{V}{O}_2\mathit{\max}$ of 10% (− 19, − 1; 95% confidence interval) compared to patients receiving adjuvant endocrine treatment (*P* = 0.028). Adjuvant radiotherapy, fatigue, age and diagnosis were not significantly associated with changes in $\dot{V}{O}_2\mathit{\max}$.

**Conclusion:**

The results of the present study indicate that adjuvant chemotherapy is associated with a subsequent reduction in $\dot{V}{O}_2\mathit{\max}$ in patients with cancer whereas MVPA before start of adjuvant treatment is positively associated with a higher $\dot{V}{O}_2\mathit{\max}$ after end of adjuvant treatment.

## Background

Althought cancer treatments have documented effects on survival, they can be invasive and toxic and cause multiple potential negative sideeffects on long term health including reduced physical functioning, impaired quality of life, chronic fatigue and reduced cardiorespiratory fitness (CRF) [1, 2].

CRF, assessed by measurement of the maximal oxygen uptake ($\dot{V}{O}_2\max \Big),$ quantifies an individual’s maximal aerobic power and provides valuable diagnostic and prognostic information about cardiovascular function, cardiopulmonary reserve, and efficiency of oxygen transport and utilization and can disclose compensatory mechanisms of abnormal cardiac function [3]. Previous studies have shown that CRF in patients with cancer is around 30% lower compared to healthy age- and sex-matched people [4–6]. In a recent meta-analysis it is implied that CRF in patients with breast cancer decreases with approximately 10% during cancer treatment and that reduced CRF can be measured even seven years after end of chemotherapy treatment [7]. These findings are of major concern given that low CRF is an important risk factor for cardiopulmonary disease and mortality in both healthy individuals and patients with cancer [8, 9]. Sufficient $\dot{V}{O}_2\max$ is related to fewer toxic effects of radiotherapy, chemotherapy, and endocrine therapy on the cardiovascular system, respiratory system, and skeletal muscles [10–15], and higher physical activity level and daily functioning in patients with cancer [16].

Physical activity (PA) is recommended as a strategy both during and after adjuvant treatment to manage treatment-related symptoms, prevent early and late co-morbidities and improve quality of life [17, 18]. Studies has shown that physical activity, especially structured training programs, can contribute to increase or maintain CRF, increase muscle strength, and improve cancer related fatigue [18, 19]. Cancer related fatigue is a common adverse effect reported in up to 99% of all cancer patients receiving adjuvant treatment in the form of radiation therapy, chemotherapy or/and biological therapy [20]. Cancer related fatigue causes a sense of physical, emotional and/or cognitive tiredness in patients which is not proportional to recent activity, and interferes with patients usual functioning [21]. Unfortunately, studies including self-reports of PA levels among patients with cancer, have shown that patients reduce their PA levels from prior to diagnosis to start of adjuvant treatment [22], and that PA levels decrease significantly during adjuvant therapy [16]. In addition, similar to healthy adults, patients with cancer are subject to the effects of ageing and age-related deconditioning that adversely affect components of the oxygen cascade and lead to reduced tolerance for exercise [13]. However, in patients with cancer, these consequences are compounded by the effects of cancer therapies leading to reductions in exercise tolerance [13].

Despite evidence that low CRF is a sign of poor prognosis for cardiopulmonary disease and mortality in both healthy individuals and patients with cancer [8, 9], there is currently little known regarding the effect of different types of adjuvant cancer treatment on $\dot{V}{O}_2\mathit{\max}$ in different cancer populations. Furthermore, the existing literature has several important methodological limitations like inconsistent assessment of physical activity across studies, prediction or determination of cardiorespiratory fitness from submaximal exercise tests, and incomplete consideration of variation of effects across population subgroups (for example, defined by BMI, age or sex), and sufficient considerations to other factors that may explain study results. Knowing the effect of different types of adjuvant cancer therapy on CRF is important to elucidate therapy-related decrements in CRF and for preventing further impairment.

Important clinical implications can arise from assessing CRF in patients with cancer, and identifying correlates associated with change in CRF. Along with data from the Phys-Can randomized-controlled trial study [23], knowledge from this study may also help to improve the specificity of exercise prescriptions to different patients with cancer based on their treatment regime. The overall long term goal would be to use this knowledge to individualize and optimize physical activity recommendations in order to maintain $\dot{V}{O}_2\mathit{\max}$ during the adjuvant phase of cancer treatment.

The primary objective of the present study was to identify correlates of changes in $\dot{V}{O}_2\mathit{\max}$ in patients undergoing adjuvant treatment for breast, prostate or colorectal cancer. More specifically, we aimed to assess whether previously described correlates (age, diagnosis, initial CRF, physical activity level), type of adjuvant treatment and cancer-related fatigue were associated with changes in $\dot{V}{O}_2\mathit{\max}$ in patients with breast, prostate or colorectal cancer.

## Methods

### Study design

The present study was a multi-center, prospective, observational cohort study investigating changes in cardiorespiratory fitness in patients undergoing adjuvant treatment for cancer without any specific exercise intervention; “The Phys-Can (Physical training and Cancer) observational study”. The included patients were intended to act as controls to those patients later recruited to the Phys-Can randomized controlled intervention study. Phys-Can has previously been described in detail [23]. Participants in the observational study received care as usual, including advice about being physically active during treatment, but were not offered to participate in an exercise intervention.

Patients aged ≥18, recently diagnosed with breast cancer, colorectal cancer or prostate cancer, scheduled for neoadjuvant chemotherapy (breast cancer) or endocrine therapy (prostate cancer), and/or adjuvant chemotherapy (breast- and colorectal cancer), adjuvant radiotherapy (breast cancer), and/or adjuvant endocrine therapy (breast and prostate cancer) or radiotherapy with curative intent without additional endocrine therapy (prostate cancer) were recruited before start of neoadjuvant/adjuvant cancer treatment. Patients who were unable to understand or express themselves in Swedish, unable to perform basic activities of daily living, showed cognitive disorders or severe emotional instability, or were suffering from other disabling comorbidity that might hamper physical exercise (e.g. unstable angina, severe heart failure, severe chronic obstructive pulmonary disease, orthopedic conditions and neurological disorders) were excluded from the study. In addition patients with breast cancer stage IIIb, men with breast cancer and patients undergoing treatment for other types of malignant disease were excluded from the study.

All patients were assessed by an oncologist or surgeon regarding eligibility to participate in the study. Patients received detailed information both written and verbally, were given ample time to consider their participation and written informed consent was obtained from each participant before entering the study. All tests were conducted before start of adjuvant therapy and again six months after baseline testing. Information on sociodemographic data were collected at baseline only.

The Phys-Can study was registered in ClinicalTrials.gov (TRN = NCT02473003, Oct, 2014).

Part of this study has been presented at The Annual Congress of the EUROPEAN COLLEGE OF SPORT SCIENCE, Prague, 2015 [24].

### Participants

Participants were recruited at the University Hospitals in Lund, Linköping and Uppsala in Sweden between September 2014 and March 2015.

**Fig. 1:**
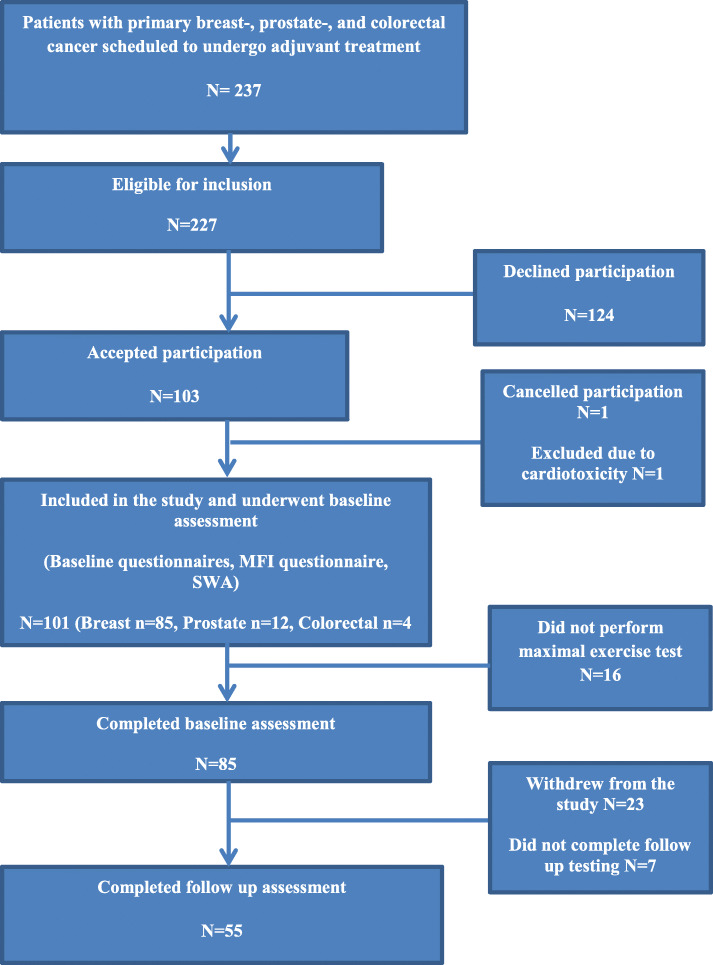
Flow-chart illustrating participant flow through enrolment, baseline measurement, and follow-up in the present study. Abbreviations: MFI, Multidimensional Fatigue Inventory-20, SWA, Sensewear Armband

Of a total of 237 patients screened, 227 were eligible for the study. Of these, 124 declined participation of various reasons, and 103 accepted participation. One participant dropped out during the inclusion process and another was excluded due to cardiotoxicity, leaving the total number included to 101 participants recently diagnosed with breast (*n* = 85), prostate (*n* = 12) or colorectal cancer (*n* = 4) (Fig. 1). Of the 101 patients included, 85 (84%) completed baseline testing and 55 (54%) completed follow-up testing. Only participants with $\dot{V}{O}_2$ max data at both baseline and post-test were included in the final analysis.

### Measures and procedures

#### Dependent variable

Cardiorespiratory fitness was assessed by maximal oxygen uptake ($\dot{V}{O}_2\mathit{\max}$) during an incremental maximal exercise test on a treadmill (Uppsala; Sports Art Fitness, TR33, Tainan, Taiwan, Linköping; GE Healthcare, T2100, USA, Lund; Rodby Innovation, RL2500E, Vänge, Sweden) according to a modified Balke protocol [23]. Participants were instructed not to eat or drink large amounts of liquids two hour before testing, refrain from coffee and nicotine two hours before test, and avoid intensive training the same day and the day prior to the test. Height (cm), weight (kg), blood pressure and heart rate at rest were measured before testing. Participants should not conduct the test if he or she: Ended cytostatic infusion less than 24 h before the test, experienced chest pains (or pressure), resting dyspnea, feeling faint or dizziness of unknown source, had injuries that could be aggravated by the test, or had a fever or infection at the time of the test. Those participants who got intravenously administered chemotherapy did the follow up CPET earliest a week after ending treatment.

Participants were fitted and familiarized to a two-way breathing mask and headgear (7450 Series V2; Hans Rudolph, Inc.) before stepping on to the treadmill and starting the test. Ventilation and gas exchange variables were measured continuously using a breath-by-breath gas analysis system (Uppsala; Sensor Medics, Vmax 29, Care Fusion, San Diego, USA) or mixing chamber (Lund and Linköping; Oxycon Pro, Erich Jaeger GmbH, Hoechberg, Germany), which was calibrated according to the instructions of the manufacturer before each test. The gas exchange variables were reported as 30 s averages. Heart rate was measured continuously with a heart rate sensor (Uppsala, Lund) (T34, Polar Electro KY, Kempele, Finland) or a 12-lead ECG recording system (Linköping) (GE Case V6.73. GE Healthcare). The rating of perceived exertion (RPE) was recorded by a standardized Borg-scale [25]. The test continued until volitional exhaustion occurred or the test leader observed indications for terminating the maximal test [26]. After completion of the test the participants walked calmly on the treadmill until ventilation, heart rate levels and leg fatigue normalized. All tests were evaluated after completion and criteria consistent with an accepted $\dot{V}{O}_2\mathit{\max}$ where set at Borg ≥17 or RER ≥1.1.

#### Independent variables

Physical activity level was objectively monitored with Sense Wear™ Mini Armband (SWA) (BodyMedia Inc. Pittsburgh, PA, USA) according to manufacturer instructions. SWA has been shown valid compared to double labeled water in healthy adults [27] and compared to indirect calorimetry in both healthy adults [28] and patients with cancer [29]. Participants were instructed to wear the SWA for seven consecutive days, and only remove it during water-based activities. Data from the SWA was downloaded and analyzed with software developed by the manufacturer (Sense Wear Professional Research Software V.8.1, Algorithm V2.2.4).

In the present study average minutes of moderate-to-vigorous intensity physical activity (MVPA) per day at baseline were included. The SWA was programmed to record PA in 1-min epochs. The cut-points defined MVPA as ≥ 3 METs according to Garber et al. [30].

Cancer related fatigue was reported using the Multidimensional Fatigue Inventory-20 (MFI) questionaire [31] at baseline. The MFI is a validated self-report questionnaire that has been used to assess fatigue in patients with a variety of cancers [32]. It consists of 20 items grouped into five subscales representing general fatigue, physical fatigue, reduced activity, reduced motivation and mental fatigue. The MFI has been reported to be a valid and reliable (average Cronbach’s alpha = 0.84) instrument for use amongst people with cancer [31]. For the present study we were interested in physical fatigue at baseline as it may hinder physical activity and in turn affect $\dot{\mathrm{V}}{\mathrm{O}}_2\max$. Cut point for determining physical fatigue was based on work by Hagelin et al. [32] and Purcell et al. [33] and were set at scores > 10.4 on the physical fatigue subscale.

Health-related data on cancer diagnosis and primary adjuvant treatment were collected from medical records at baseline.

Sociodemographic data on age, sex, education, work situation, living conditions, and sick leave was collected from study-specific questionnaires.

### Statistical analysis

Descriptive characteristics are presented as mean values with standard deviation (SD) unless otherwise stated and results as mean with 95% confidence intervals (CI).

Between-group differences were analyzed with Wilcoxon Signed Ranks test due to lack of assumption about normality and homogeneity of variance.

Multivariate linear regression models were applied to assess whether age, diagnosis group, type of adjuvant treatment, MVPA at baseline and physical fatigue at baseline were associated with changes in $\dot{V}{O}_2\mathit{\max}$. Diagnosis group was recoded into two categories; “breast cancer” and “prostate and colorectal cancer” due to few participants in the two latter groups. Type of adjuvant treatment was coded into three categories; “adjuvant chemotherapy treatment”, “radiotherapy treatment” and “endocrine therapy treatment”. Physical fatigue at baseline was coded as “physical fatigue at baseline” or “no physical fatigue at baseline”.

The final multivariate model was built as described by Hosmer and Lemeshow [34]. This means that all independent variables with *P* ≤ 0.2 in the bivariate analysis were included in the multivariate analysis. Adjuvant radiation therapy was included in the multivariate analysis despite *P* ≥ 0.2 because type of adjuvant treatment was recoded into three design variables and adjuvant chemotherapy treatment had a *P* ≤ 0.2. Also, type of adjuvant treatment is known to be a clinically important variable for $\dot{V}{O}_2\mathit{\max}$ in breast cancer patients [8]. In the multivariate analysis the least significant variable from the bivariate analysis was removed in a step-wise fashion and the analyses were performed again. This removed age and no physical fatigue. The procedure was repeated until only significant independent variables remained. Adjuvant treatment and MVPA were not removed from the final model.

The IBM SPSS version 22.0 statistical software program (Statistical Product and Service Solutions, Chicago, IL, USA) was used for all analysis. The level of significance was set to 0.05.

## Results

Table 1 shows the characteristics of the study participants at baseline stratified by $\dot{V}{O}_2\mathit{\max}$ measurement at both baseline and six months compared to baseline only.

**Table 1:** Baseline characteristics of participating subjects with (*n* = 55) and without (*n* = 30) maximal oxygen uptake measurements before and six months after start of adjuvant cancer treatment. Results are presented as frequencies and percentages in parenthesis unless otherwise stated*

	Baseline and follow up *n* = 55 (64.7)	Baseline only *n* = 30 (35.3)
Age yrs., mean (SD)	58.8 (11.1)	59.2 (12.9)
Height cm, mean (SD)	168 (7.7)	167 (5.6)^a^
Weight kg, mean (SD)	74.3 (14)	75.2 (16)^a^
Females, n (%)	42 (80.8)^b^	27 (90)
Cohabitation	b	
Married/cohabitant, n (%)	42 (80.8)	21 (70)
One couple, two households, n (%)	3 (5.8)	3 (10)
Living alone, n (%)	7 (13.5)	6 (20)
Education level, n (%)	c	
Compulsory school, n (%)	4 (7.8)	4 (13.3)
High School, n (%)	17 (33.3)	8 (26.7)
College/university n (%)	30 (58.8)	18 (60)
Working status	b	
Full time work, n (%)	15 (28.8)	6 (20)
Part time work, n (%)	6 (11.5)	3 (10)
Retired, Homemaker n (%)	20 (38.5)	12 (40)
Sick leave, n (%)	11 (21.2)	9 (30)
Diagnosis		
Breast cancer, n (%)	45 (81.8)	26 (86.7)
Prostate cancer, n (%)	8 (14.5)	2 (6.7)
Colorectal cancer, n (%)	2 (3.6)	2 (6.7)
Primary adjuvant treatment		
Chemotherapy, n (%)	20 (36.4)	14 (46.7)
Type chemotherapy^d^		
Docetaxcel + FEC low-dose	9 (45.0)	
Docetaxcel + FEC high-dose	8 (40.0)	
CAPOX	1 (5.0)	
Capecitabine single	1 (5.0)	
Radiation therapy, n (%)	18 (32.7)	10 (33.3)
Endocrine treatment, n (%)	17 (30.9)	6 (20)
Physical fatigue, n (%)	47 (94)^e^	28 (93.3)
MVPA (SD)* (min^.^day^−1^)	47.6 (30.1)^f^	33.3 (19.5)^g^

Abbreviations: Docetaxcel, breast cancer only, low-dose 75–80 mg/m^2^, high-dose 90–100 mg/m^2^. FEC, breast cancer only, low to high-dose, fluorouracil 500–600 mg/m^2^ and/or only Epirubicin 75–100 mg/m^2^ and cyclophosphamide 500–600 mg/m^2^. CAPOX, colorectal only, Capecitabine and Oxaliplatin. Capecitabine single, colorectal only. *MVPA*, moderate and vigorous physical activity

^a^ Missing = 5

^b^ Missing = 3

^c^ Missing = 4

^d^ Missing = 1, % of n participants receiving chemotherapy

^e^ Missing = 5

^f^ Missing = 7

^g^ Missing = 24

Participants with and without follow up $\dot{V}{O}_2\mathit{\max}$ measurements were similar with respect to age, height, weight, cohabitation, education level, working status and diagnosis. Physical fatigue was reported by 94% of participants at baseline. There were no differences in MVPA levels between diagnosis groups at baseline.

In linear regression analysis, fatigue, age and diagnosis were not associated with changes in $\dot{\ V}{O}_2\mathit{\max}$ during treatment. MVPA at baseline and adjuvant treatment explained 23% of the variability in changes in $\dot{V}{O}_2\mathit{\max}$ (Table 2).

**Table 2:** Regression summaries for multivariate analysis with the dependent variable % change in VO2max from before treatment to six months follow up. The coefficients are given with 95% confidence intervals

	Step-wise multivariate analysis		Final multivariate analysis	
	Coefficients	*P*-values	Coefficients	*P*-values
Breast cancer	8.829 (−4.085, 21.743)	0.174		
Adjuvant chemotherapy treatment*	−8.591 (− 18.959, 1.778)	0.102	− 10.066 (− 19.000, − 1.131)	0.028
Adjuvant radiation therapy treatment *	−1.116 (− 12.426, 10.194)	0.843	−3.490 (− 12.870, 5.890)	0.457
MVPA at baseline (min^.^day^− 1^)	0.182 (0.036, 0.327)	0.016	0.167 (0.042, 0.291)	0.010
Age (yrs)	0.075 (−0.473, 0.623)	0.783		
No physical fatigue at baseline	3.098 (−17.323, 23.519)	0.760		

Abbreviations: MVPA; Moderate- to-vigorous physical activity; VO2max; maximal oxygen uptake

*Compared to endocrine therapy treatment

A 30 min higher MVPA before start of adjuvant treatment was associated with a 5% higher $\dot{V}{O}_2\mathit{\max}$ (*P* = 0.010) at six months follow up when adjusted for adjuvant treatment (Table 2). Adjuvant chemotherapy treatment was associated with a reduction in $\dot{V}{O}_2\mathit{\max}$ (*P* = 0.028). Patients receiving adjuvant chemotherapy treatment had a mean decline in $\dot{V}{O}_2\mathit{\max}$ of 10% (− 19, − 1.1, 95% CI) compared to patients receiving adjuvant endocrine therapy treatment only. Adjuvant radiotherapy and endocrine therapy were not associated with changes in $\dot{V}{O}_2\mathit{\max}.$

Table 3 shows average $\dot{V}{O}_2\mathit{\max}$, peak $\dot{V}$E, HR, RER and RPE from the maximal exercise test at baseline and six months after start of adjuvant treatment for the different treatment groups. There was a significant increase (*P* = 0.031) in RPE peak from before treatment to six month follow up, from 16.6 to 17.2, respectively in the radiotherapy group. There were no significant changes in $\dot{V}{O}_2\mathit{\max}$, $\dot{V}$ E peak, RER peak, or HR peak.

**Table 3:** Data from the exercise test of the study participants who completed baseline and follow up testing (*n* = 55) presented by before treatment and after six month follow up, stratified by treatment group. Data are given as mean with confidence intervals (CI) and standard deviation (SD) in parentheses unless otherwise stated*

		Before treatment mean (SD)	95% CI	Six month follow up mean (SD)	95% CI	*P-*value^b^
Chemotherapy (*N* = 20)	VO2max (ml kg^−1^. min^− 1^)	31 (7.1)	27.6–34.3	28.7 (6.2)	25.8–31.6	.191
	VE peak (L min^− 1^)	84 (15.4)	76.8–91.2	82.4 (19.4)	73.3–91.5	.296
	RER peak	1.25 (0.1)	1.22–1.29	1.22 (0.1)	1.17–1.27	.147
	RPE peak	17.2 (1.2)	16.6–17.8	17.8 (1.2)	17.2–18.3	.058
	HR peak (beats. min^−1^)	173 (13)	166.5–178.6	170 (11.1)	164.3–174.6	.054
Radiotherapy (*N* = 18)	VO2max (ml kg^−1^. min^− 1^)	29.4 (6.7)	26.1–32.7	29.4 (7.9)	25.5–33.3	.794
	VE peak (L min^− 1^)	77.8 (18.7)	68.5–87.1	73.5 (28.2)	59.4–87.5	.845
	RER peak	1.20 (0.1)	1.15–1.25	1.17 (0.1)	1.12–1.23	.140
	RPE peak	16.6 (1.3)	15.9–17.2	17.2 (1.5)	16.4–17.9	.031*
	HR peak (beats. min^−1^)	169 (20.4)	158.6–178.9	165 (16.4)	156.7–173	.138
Endocrine therapy (*N* = 17)	VO2max (ml kg^− 1^. min^− 1^)	32 (6.8)	28.5–35.5	33.1 (9.2)	28.4–37.8	.162
	VE peak (L min^− 1^)	86.6 (18.6)	77.1–96.2	83.7 (17.2)	74.8–92.5	.113
	RER peak	1.23 (0.1)	1.16–1.30	1.19 (0.1)	1.13–1.24	.162
	RPE peak	17.4 (1.2)	16.8–18	17.2 (1.7)	16.3–18.1	.558
	HR peak (beats. min^−1^)	166 (17.8)	156.9–175.2	166 (19.7)	156.2–176.4	.816

^a^ Abbreviations: VO2max, highest recorded oxygen uptake during exercise test; VE peak, highest recorded ventilation; RER peak, highest recorded respiratory exchange ratio; RPE peak, highest reported perceived exertion; HR peak, highest recorded heart rate; MVPA, Moderate and vigorous physical activity

^b^ *P*-values for any differences between groups

## Discussion

The results of the present study provide knowledge about clinically relevant correlates to subsequent changes in cardiorespiratory fitness in patients with cancer during adjuvant treatment. Findings indicate that adjuvant chemotherapy treatment is associated with a 10% decline in $\dot{\ V}{O}_2\mathit{\max}$ six months after start of treatment. The results also indicate that time spent in MVPA before start of adjuvant treatment is associated with higher levels of $\dot{V}{O}_2\mathit{\max}$ six months later. Fatigue, age and diagnosis were not associated with changes in $\dot{V}{O}_2\mathit{\max}$ during treatment.

Our results are in line with the work of several other studies examining the association between cancer treatment and cardiorespiratory fitness [5, 8, 35, 36]. In a recent meta-analysis including 26 clinical trials and observational studies that measured $\dot{V}{O}_2\mathit{\max}$ pre- or post- adjuvant therapy, it was shown that CRF in patients with breast cancer decreased with approximately 10% during cancer treatment and that reduced CRF can be measured seven years after end of chemotherapy treatment [7]. In the study by Peel et al. [7], where 78% of the women were treated with adjuvant chemotherapy, patients with breast cancer had a 17% lower weighted mean $\dot{V}{O}_2\mathit{\max}$ prior to adjuvant treatment and a 25% lower $\dot{V}{O}_2\mathit{\max}$ after completion of adjuvant therapy compared to healthy sedentary women [7]. In the present study, women treated with adjuvant chemotherapy had a similar $\dot{V}{O}_2\mathit{\max}$ prior to adjuvant treatment as healthy age matched women (30.4 ml kg^− 1 **.**^ min^− 1^) [37], but a 8.5% lower $\dot{V}{O}_2\mathit{\max}$ (27.9 (ml kg^− 1 **.**^ min^− 1^) six months after start of adjuvant chemotherapy treatment compared to healthy age matched women. Our patients had a higher $\dot{V}{O}_2\mathit{\max}$ in both the pre- and post-adjuvant setting compared to the patients in the study by Peel et al. [7], which could be due to differences in testing protocol (treadmill vs. bicycle), but the relative decline of 10% was similar.

The reason for the decline in $\dot{V}{O}_2\mathit{\max}$ in patients treated with adjuvant chemotherapy is likely multifactorial, involving multiple organ components of the oxygen transport chain [7]. Many of these negative effects of chemotherapy are dose dependent, but although treatment regimens are focusing on reducing doses of chemotherapy, there are still several aspects which could reduce $\dot{V}{O}_2\mathit{\max}$ both directly and indirectly, through reduced physical activity levels. Chemotherapy used in breast cancer treatment are associated with both short- and long term cardiac complications, and anthracycline-based adjuvant therapy and treatment with trastuzumab in particular, carries a substantial long term risk of heart failure [4, 36]. Unfavorable alterations in myocardial tissue due to chemotherapy treatment results in reduced left ventricular ejection fraction which in turn reduces convective oxygen delivery [13]. Also, anthracycline-caused cytotoxic damage leads to compensatory alterations in autonomic tone, which may have implications for the heart rate reserve (HRR) and CRF [38]. However, we did not see any unfavorable changes in heart rate in our patients**.** Except for the radiotherapy group, all patients reported consistent RPE at both pre- and posttest indicating the same amount of effort. Furthermore, studies have reported negative effects of chemotherapy directly to skeletal muscle [39, 40]. In these studies, both the force-generating abilities and mitochondrial function are affected negatively and the decrease is dependent upon the length of chemotherapy exposure [39, 40].

It has previously been reported that patients reduce their PA levels prior to adjuvant treatment [22], and that $\dot{V}{O}_2\mathit{\max}$ in cancer patients are already lower prior to adjuvant treatment compared to healthy age matched controls [7]. Our patients had a mean MVPA of 47.6 (30.1) min^.^day^− 1^ prior to adjuvant treatment, which is above the minimum recommended 150 min of moderate intensity PA per week [17]. However, mean MVPA varied among the participants. We observed that those with mean MVPA less than 30 min per day before start of adjuvant treatment had a lower mean $\dot{V}{O}_2\mathit{\max}$ both prior and after treatment compared to those with mean MVPA > 30 min (results not shown). The group with < 30 min MVPA per day were also the only group with a significant decline in $\dot{V}{O}_2\mathit{\max}$ from before start of treatment to six months after. A study on PA among German patients with breast cancer found that PA levels decreased significantly during adjuvant therapy, and the decline was stronger in patients treated with chemo- and/or radiotherapy compared to endocrine or no adjuvant therapy [16]. This corresponds to our findings were adjuvant chemotherapy were associated with a decline in $\dot{V}{O}_2\mathit{\max}$, possibly due to a reduced PA level in addition to the possible cardiac and direct muscular complications. Alternatively, our results might indicate that a minimum threshold of MVPA before and during treatment can protect against some of the side effects normally observed with adjuvant chemotherapy.

There is evidence that physical activity, even high intensity exercise, is both feasible and safe for patients with cancer both during and after adjuvant treatment [17, 18]. Patients with cancer participating in exercise programs may increase or maintain their CRF [18], and also, there is evidence that exercise can protect against the acute cardiotoxic effect of anthracyclines and in turn, reduced convective oxygen delivery [41]. Our findings suggest that it could be beneficial to take measures to promote PA to patients with cancer before start of oncological treatment to prevent a decrease in PA level, and consequently a reduced $\dot{V}{O}_2\mathit{\max}$. Studies have shown that an oncologist recommendation [42] and physical activity facilities in connection with the hospital [43], might be of importance to increase exercise behavior in newly diagnosed patients.

The present study had a number of strengths, including the inclusion of patients with different cancer diagnosis and type of treatment, the objective measurements of physical activity and direct and maximal testing of cardiorespiratory fitness before start of adjuvant treatment. To our knowledge, few studies have included measured $\dot{V}{O}_2\mathit{\max}$ in patients with different cancer diagnosis before start of adjuvant therapy [44].

The present study also had several limitations that should be noted. Less than 50% of the eligible patients completed the study. There was a larger proportion of women and patients with breast cancer among the included patients, and results should be interpreted with caution for patients with colorectal or prostate cancer. Although we did our best to recruit patients with prostate cancer and colorectal cancer, we experienced significant problems in recruiting the two latter groups. Despite the overrepresentation of patients with breast cancer, the distribution between the different cancer therapies (chemotherapy, radiotherapy and endocrine therapy) was evenly distributed, with around 30% in each treatment group. Our sample consists of patients who are sufficiently fit to participate in such a study, and might be more active than the average patient group resulting in higher $\dot{V}{O}_2\mathit{\max}$ values [45]. However, in case of such a bias, real PA and CRF in the overall population of patients with cancer may be even lower than in our study.

## Conclusion

Adjuvant chemotherapy was associated with a 10% reduction in $\dot{V}{O}_2\mathit{\max}$ during treatment whereas higher levels of MVPA before start of adjuvant treatment were positively associated with a higher $\dot{V}{O}_2\mathit{\max}$ after end of adjuvant treatment. As there was a larger proportion of women with breast cancer among the included patients, we cannot generalize these results to all cancer populations. However, these results combined with previous findings of impaired $\dot{V}{O}_2\mathit{\max}$ among patients with cancer [46–49], emphasize the clinical importance of increasing or maintaining $\dot{V}{O}_2\mathit{\max}$ in this phase of cancer treatment, and highlight the importance of physical activity on cardiorespiratory fitness in patients with cancer. Future studies should address increasing PA in the early phase of treatment and further examine the association between PA and $\dot{V}{O}_2\mathit{\max},$ and in particular the underlying mechanisms.

## Data Availability

The datasets used and/or analysed during the current study are available from the corresponding author on reasonable request.

## Abbreviations

RER: Respiratory exchange ratio
Borg RPE Scale: Borg Ratings of Perceived Exertion Scale
Hb: Hemoglobin
HR: Heart rate
HRR: Heart rate reserve
$$ \dot{V}\mathrm{E} $$: Minute ventilation
$$ \dot{V}{O}_2 $$: Oxygen uptake
$\dot{V}{O}_2\mathit{\max}$: Maximal oxygen uptake
CPET: Cardiopulmonary exercise test
CRF: Cardiorespiratory fitness
PA: Physical activity
SWA: SenseWear Armband
MVPA: Moderate-to-vigorous intensity physical activity
METs: Metabolic equivalent tasks
